# The Imbalance of Homocysteine, Vitamin B12 and Folic Acid in Parkinson Plus Syndromes: A Review beyond Parkinson Disease

**DOI:** 10.3390/biom14101213

**Published:** 2024-09-26

**Authors:** Vasiliki Poulidou, Ioannis Liampas, Marianthi Arnaoutoglou, Efthimios Dardiotis, Vasileios Siokas

**Affiliations:** 1 First Department of Neurology, AHEPA University Hospital, Aristotle University of Thessaloniki, Stilponos Kyriakidi 1, 54636 Thessaloniki, Greece; basia_poulidou@yahoo.gr; 2 Department of Neurology, University Hospital of Larissa, School of Medicine, University of Thessaly, 41100 Larissa, Greece; liampasioannes@gmail.com (I.L.); edar@med.uth.gr (E.D.); 3 Department of Clinical Neurophysiology, School of Medicine, AHEPA University Hospital, Aristotle University of Thessaloniki, Stilponos Kyriakidi 1, 54636 Thessaloniki, Greece; marnaoutoglou@yahoo.com

**Keywords:** homocysteine, vitamin B12, folate, Parkinson plus syndromes, alpha-synuclein, tau protein

## Abstract

While there is a link between homocysteine (Hcy), B12 and folic acid and neurodegeneration, especially in disorders like Parkinson’s and Alzheimer’s diseases, its role in Parkinson plus syndromes (PPS) has only been partially investigated. It appears that elevated Hcy, along with an imbalance of its essential vitamin cofactors, are both implicated in the development and progression of parkinsonian syndromes, which represent different disease pathologies, namely alpha-synucleinopathies and tauopathies. Attributing a potential pathogenetic role in hyperhomocysteinemia would be crucial in terms of improving the diagnostic and prognostic accuracy of these syndromes and also for providing a new target for possible therapeutic intervention. The scope of this review is to focus on vitamin imbalance in PPS, with a special emphasis on the role of Hcy, B12 and folic acid in the neurodegenerative process and their implication in the therapeutic approach of these disorders.

## 1. Introduction

The contribution of homocysteine (Hcy) to the development of neurodegenerative disorders has been increasingly recognized. Vitamins involved in Hcy metabolism, including vitamin B12 and folate (vitamin B9), also participate in the neurodegenerative process [1]. The incidence of neurodegenerative diseases increases with advanced age, as do Hcy levels [2]. Accumulating evidence favors the view that Hcy has a neurotoxic effect on disorders such as Alzheimer’s disease (AD) [3,4,5] and Parkinson’s disease (PD) [6,7,8,9]. This is not surprising since vitamins of the B complex are essential in the neurogenesis, neurotransmission and maintenance of homeostasis within the body and brain [10]. In spite of the fact that this evidence may not be generalizable to Parkinson plus syndromes (PPS), it can be assumed that these neurodegenerative atypical parkinsonian syndromes, which share overlapping clinical and histopathological similarities with both AD and PD are also linked to Hcy imbalance.

Although the dysregulation of Hcy metabolism has been intensively investigated in PD, the same does not apply to PPS. This contradiction could be explained by several reasons: Firstly, PPS represent a family of less common disorders in comparison to PD, resulting in small sample sizes in observational cohorts. Secondly, decreased vitamin B12 levels and increased Hcy are more commonly encountered among levodopa-treated patients [11], and this imbalance has been especially investigated in the context of intestinal levodopa/carvidopa infusion therapy [12], an advanced treatment reserved for PD but not for PPS patients. Interestingly, concentrations of total Hcy in PD patients were observed to be higher following the initiation of L-dopa therapy than before treatment in both serum and CSF, attributing a potentially causative role to L-dopa [13,14]. In addition, lower vitamin B12 levels might be more likely to develop in PD because some levodopa-treated patients adopt a low-protein diet, which results in an alteration in dietary intake of vitamin B12 [1]. Gastrointestinal dysfunction, which can manifest as constipation, delayed gastric emptying and Helicobacter pylori infection, all features of PD, can also be linked to the higher frequency of vitamin B12 deficiency in PD patients [15].

## 2. Homocysteine in Health and Disease

Hcy is an intermediate amino acid that is generated during the metabolism of methionine to cysteine. Vitamins such as Vitamin B6, folate and Vitamin B12 are mandatory cofactors for both pathways of Hcy metabolism: vitamin B6 for transsulfuration of Hcy to cystathionine and vitamin B12 and folate for Hcy to remethylate to methionine [16]. The processes of transsulfuration and remethylation are of crucial importance for the balance of Hcy metabolism and thus for circulating levels of Hcy [17]. Therefore, an important interaction between Hcy, vitamin B12 and folate is established [18]. Methylmalonic acid (MMA), an intermediate metabolite in vitamin B12 metabolism is also implicated in these interactions and can be increased in vitamin B12 deficiency but not in folate deficiency. In the absence of vitamin B12, the conversion of methylmalonyl-CoA to succinyl-CoA cannot proceed normally and serum MMA accumulates [19]. Hcy can also be converted into homocysteine thiolactone which enhances the production of reactive oxygen species (ROS) and, ultimately, the initiation of processes leading to oxidative stress [20]. Increased levels of Hcy, a condition called hyperhomocysteinemia (HHcy), can result from either deficiency of the aforementioned vitamin cofactors or from variants in genes encoding the key enzymes of the Hcy metabolic cycle [17]. Vitamin B12 deficiency can result in HHcy and neurodegeneration [21]. On the other hand, the supplementation of vitamins results in lower Hcy levels [22]. Various pathological conditions, such as diabetes mellitus, hypertension, renal insufficiency, alcohol abuse, reduced physical activity and advanced age can also be associated with HHcy [23]. The definition of HHcy includes plasma levels above 15 μmol/L, with separate subcategorizations as follows: moderate (15 and 30 μmol/L), intermediate (30 and 100 μmol/L) and severe HHcy (above 100 μmol/L) [24].

### Homocysteine and Neurodegenerative Diseases

Hcy is implicated in the pathogenesis of neurodegenerative disorders in multiple ways, especially via mediating inflammatory response [25]. Oxidative stress is an established factor that contributes to neurodegeneration [26]. Activation of N-methyl-d-aspartate (NMDA) receptors by Hcy, a potent agonist of these receptors, can result in calcium overload and glutamate excitotoxicity in the cortical neurons [27,28,29]. These processes are thought to contribute significantly to Hcy neurotoxicity. The DNA damage and triggering of apoptosis are other mechanisms involved in the neurotoxicity caused by Hcy. Indeed, several studies have demonstrated the deleterious effect of Hcy to neurons by eliciting excessive cell dysfunction and accumulated DNA damage [27,30]. Moreover, Hcy has been shown to exert a noxious effect on substantia nigra neurons [31]. Elevated Hcy can facilitate pathological aggregation of proteins involved in neurodegenerative disorders and be toxic to dopaminergic neurons [1,32].

**Figure 1:**
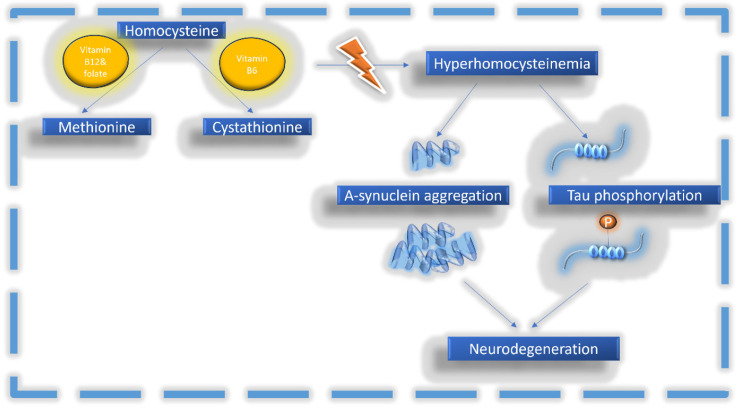
Hcy metabolism is mediated by vitamin cofactors. HHcy can facilitate the pathological accumulation of α-synuclein and increase the levels of phosphorylated tau.

HHcy appears to be implicated in the neurodegeneration process of both the alpha synucleinopathies and the tauopathies, which represent the two groups of different pathophysiologies of PPS. The proposed mechanisms responsible for this interaction are shown in Figure 1, and the results from the available studies will be discussed further in the following sections.

## 3. Parkinson Plus Syndromes

PPS represent a group of neurodegenerative disorders with different underlying pathology, which exhibit some of the fundamental clinical features of idiopathic PD [33,34]. The initial clinical presentation of PPS may resemble idiopathic PD making an early diagnosis quite difficult [33]. However, with disease progression, other symptoms such as early dementia, visual hallucinations, prominent autonomic failure, pyramidal signs, gait disturbance with early falls, ophthalmoparesis and cortical sensory deficits help distinguish different disorders. It is important to differentiate PPS from PD because of differences in prognosis and treatment [33]. One of the primary features that clinically distinguishes PPS from PD is the lack of a sustained response to levodopa therapy. In contrast to the symptomatic benefit of levodopa in PD, an absent or poor response to levodopa is a characteristic feature of PPS [33].

The pathophysiology of PPS can be categorized into two groups: tauopathies and alpha-synucleinopathies. The aforementioned groups of neurodegenerative proteinopathies share similar mechanisms of propagation, resembling Prion-like propagation [35,36,37,38].

The alpha synucleinopathies represent a group of disorders characterized by abnormal aggregation of α-synuclein (α-syn) in the cytoplasm of neuronal or glial cells [39]. A study of synucleinopathy animal PD models indicates that α-syn causes upregulation of DNA damage response [40]. Synucleinopathies are represented by PD, Dementia with Lewy Bodies (DLB) and Multiple System Atrophy (MSA). Between these three disorders, significant pathological differences exist, including the cell type involved and the amount of neuronal loss. Specifically, neuronal cells are involved in PD and DLB and oligodendrocytes in MSA, suggesting that MSA is a primary disorder of the glia. In addition, neuropathology affects only selected regions in PD but follows a more widespread pattern throughout many regions in MSA [39]. The involvement of certain genes like leucine-rich repeat kinase 2 (*LRRK2*) and *parkin*, which are pathogenic for PD, has also been investigated in other synucleinopathies, like MSA. The study by Huang et al. has shown that *LRRK2* expression is increased in the early stages during the disease processes of MSA, before the formation of the characteristic glial cytoplasmic inclusions [41].

The tauopathies result from abnormal phosphorylation of tau, a protein involved in various cell processes, namely axonal transport and stabilization of neuronal microtubules [42]. Under abnormal circumstances, tau aggregates into neurofibrillary tangles, interfering with microtubule function and impairing axonal transport. In normal cells, 3R-tau and 4R-tau (isoforms with either three or four repeats of the tau microtubule-binding domain) occur in approximately equal proportions. The disarrangement of that normal ratio is considered to lead to neurodegeneration [42]. Tauopathies are thus subclassified by whether tau inclusions are predominantly made of 3R or 4R tau, or a mixture of both. From a clinical point of view, tauopathies can cause a range of phenotypes from neurodegenerative dementias to atypical Parkinsonism syndromes and the main representations encompass Frontotemporal Dementia (FTD), AD, progressive supranuclear palsy (PSP) and corticobasal degeneration (CBD) [43].

### 3.1. Synucleinopathies

A-syn is a protein expressed in neuronal cells and is mainly localized at presynaptic terminals. Under normal circumstances, it has an important role in controlling protein and neurotransmitter release. Abnormal conditions, like increased oxidative stress, can trigger irregular aggregation of α-syn and lead eventually to the formation of Lewy bodies, the pathologic hallmark of diseases such as PD, DLB and MSA [44].

Hcy can facilitate abnormal aggregation of proteins such as α-syn and ultimately act deleteriously on dopaminergic neurons [10,32]. A recent study in an animal PD model concluded that higher Hcy levels correlate with more profound α-syn deposition within mouse brains and that N-homocysteinylation of α-syn contributes to its accumulation and neurotoxicity [45]. Specifically, Hcy modifies α-Syn to form more toxic fibrils, which are resistant to digestion by proteinases and exhibit increased propagation activity [45,46]. In contrast, blocking N-homocysteinylation alleviates α-syn pathology and decreases the loss of dopaminergic neurons implying that this strategy could be used as a novel therapeutic approach against PD [45]. In this direction, other studies suggest that vitamin B12 could potentially prevent α-syn-mediated neurodegeneration through different mechanisms. For example, vitamin Β12 inhibited α-syn fibrillogenesis and alleviated the cytotoxicity of α-syn aggregates in a recent study [47] and has also exhibited neuroprotective properties through the modulation of *LRRK2*, leading to the prevention of α-syn accumulation and alleviation of dopaminergic dysfunction in another study [48]. This is an important observation because apart from being involved in PD, there is growing evidence that *LRRK2* may play a role in the disease pathogenesis of MSA as well [41,49,50]. These results could be promising in regard to the hypothesis that vitamin supplementation may influence the risk of the development of PD and PPS, although further studies are needed. The results of the studies providing evidence of the Hcy and vitamin dysregulation in alpha-synucleinopathies are summarized in Table 1.

#### 3.1.1. Multiple System Atrophy

MSA is the third main a-synucleinopathy, with some crucial clinical and pathological differences compared to PD and DLB. MSA is a unifying term for a disorder that encompasses signs and symptoms of parkinsonism, autonomic failure and cerebellar syndrome in various combinations [61]. Glial cytoplasmic inclusions represent the pathologic hallmark of MSA and consist of cytoplasmic aggregates of alpha-synuclein (Lewy bodies) in oligodendrocytes. As mentioned above, the involvement of glial cells distinguishes MSA from other synucleinopathies such as PD, in which alpha-synuclein aggregates are present in neuronal cells [61].

The latest criteria for the diagnosis of MSA were revised in 2022. In these criteria, the diagnosis of clinically established MSA requires brain MRI findings. Also, a category of possible prodromal MSA was included only for research purposes in order to identify patients in the earliest stages [62].

Several studies have examined whether elevated Hcy levels may be associated with MSA with the aim of exploring the impairment in the regulation of vitamin homeostasis in MSA patients, as well as their potential role as biomarkers [52,53,54,55,60]. Most of the studies have proved that MSA patients have significantly higher levels of serum Hcy compared with healthy subjects, and this elevation is seen especially in male patients. In one study that explored the metabolic derangements in patients diagnosed with PD, there were no statistically significant differences in serum Hcy levels in patients with PD compared with patients with other parkinsonian syndromes such as MSA and PSP, but no comparison was made with healthy controls [59]. A recent meta-analysis which was based on three of these studies concluded that serum Hcy levels are higher in MSA patients than in normal controls [63].

Zhang et al. demonstrated that MSA patients have higher levels of Hcy compared to healthy controls and that the increased Hcy level is more frequently observed in male than in female patients. However, the levels of vitamin B12 and folate were not statistically different between the two groups [52]. McCarter and colleagues have recently examined whether vitamin B12 levels measured at MSA diagnosis are associated with total survival in these patients [64]. Indeed, shorter survival time is associated with lower vitamin B12 levels at the time of diagnosis and vice versa, longer survival is observed when vitamin B12 levels are increased. Another case–control study in a large MSA cohort revealed important alterations in blood vitamins of MSA patients, a finding that underscores that dysregulation of vitamin homeostasis has a pivotal role in the neurodegenerative process of MSA. In particular, MSA patients exhibited significantly decreased serum folate levels in comparison with healthy subjects, while no significant differences were found in serum vitamin B12 concentrations among the two groups [60]. Inadequate intake and malabsorption of folate due to gastrointestinal dysfunction, an important feature of MSA, may explain such deficiency [65]. Apart from vitamin B, this study showed that serum levels of vitamins A and C were significantly higher in MSA patients compared to controls. A previous cross-sectional study has revealed a possible connection between vitamin C and Hcy levels, suggesting that vitamin C can act as a scavenger for free radicals formed by Hcy and may be related to plasma levels of Hcy [66]. Another study concluded that in terms of estimating the severity of the disease, Hcy was the most valid and reliable among the inflammatory mediators [53]. A pronounced elevation in the levels of Hcy and a decrease in the levels of uric acid (UA) was revealed in patients with MSA in comparison to normal subjects, implicating once more that oxidative stress caused by Hcy plays a pivotal role in the disease development and also contributes to the pathophysiological mechanisms involved in MSA [53]. These changes were more pronounced in the male population of the study. Interestingly, the combination of Hcy and UA could discriminate MSA patients from normal controls implying that this new screening diagnostic instrument could be used to increase the diagnostic accuracy in MSA patients [53]. However, another study failed to find any significant changes in the UA concentrations in patients with MSA [54]. The combination of Hcy with other inflammatory mediators (namely serum Klotho and vitamin D) has also been investigated as a potential diagnostic biomarker for differentiating MSA patients from controls, as well as male MSA patients from male PD patients [55]. Apart from elevated serum levels of Hcy in MSA patients, decreased serum levels of Klotho and vitamin D were also observed compared to healthy subjects. Again, these observations were found especially in male patients. Secondly, serum levels of these inflammatory mediators were remarkably correlated with the severity of MSA [55]. The results of another study, which aimed to explore whether oxidative stress could be implicated in the development of MSA, showed that apart from proinflammatory agents such as Hcy, the levels of which were elevated, levels of agents with antioxidant capacity, such as bilirubin, were decreased in MSA patients [54].

#### 3.1.2. Dementia with Lewy Bodies

Following AD, DLB is the second most common type of degenerative dementia. Lewy body dementia (LBD) is an umbrella term that includes PD with dementia and DLB [67]. Dementia is an essential clinical feature of the diagnosis of DLB and is often the presenting symptom [68]. The fundamental clinical features also include parkinsonism, fluctuations in cognition, visual hallucinations and REM sleep behavior disorder [68]. The most recent criteria for DLB were revised in 2017 to improve diagnostic sensitivity and specificity [68].

It is already known that DLB is associated with neuropathologic changes that overlap with PD and AD [69]. Firstly, Lewy bodies are the pathologic hallmark of DLB and they are present throughout the neocortex as well as in the brainstem nuclei and limbic structures [68]. Secondly, concomitant AD neuropathologic changes can be observed in many DLB patients [70,71]. Interestingly, Alzheimer-related lesions can impact the progression of the neurodegenerative process and DLB patients with mixed pathologies of amyloid beta, tau and alpha-synuclein typically exhibit a more severe cognitive decline [72,73]. In addition, some neuropathologic data imply a concomitant presence of vascular disease pathology in patients with DLB [72,74,75]. From a clinical perspective, cognitive impairment is significantly associated with low vitamin B12, high MMA and high Hcy in older adults [76,77]. Previous randomized controlled studies in patients with Mild Cognitive impairment (MCI) showed that treatments targeting Hcy (like vitamins of the B complex) can decrease the rate of brain atrophy and cognitive decline and that those with the highest baseline level of Hcy have the greatest treatment effect [78,79]. In conclusion of the above and taking into consideration that HHcy is an already-known risk factor for MCI, AD and vascular disease, it can be assumed that it can also play a role in DLB.

There is a lot of evidence supporting the above assumption. In a recent case–control study, which included DLB, AD patients and healthy control, it was proven that increased plasma Hcy and decreased serum folate levels were independently associated with DLB and that the association of raised Hcy was more prominent for DLB than for AD [56]. Whether Hcy plays a causative role in DLB remains to be elucidated since no relationship between Hcy levels and symptom duration was observed in this study [56]. The results of another study were conflicting since recently diagnosed AD patients were shown to have lower levels of vitamins B1, B6, B12 and folate and higher Hcy levels in serum compared to controls, but as far as the DLB patients were concerned only vitamin B12 was reduced [57]. However, DLB patients showed decreased folate and vitamin B12 concentrations, similar to AD patients. In another study, significantly higher levels of Hcy and lower levels of vitamin B12 were observed in different subtypes of dementia, including DLB [58]. Nevertheless, no significant relevance was found between serum Hcy, folate and vitamin B12 levels, cognitive function scales and MRI markers in the DLB group. Lovati et al. investigated the serum folate levels in patients suffering from cortical and subcortical dementia, including LBD, CBD and PSP. Although a trend towards lower levels of folate was found in LBD and CBD, this result was not statistically significant [51]. The value of this result is questionable, mainly because of the limited number of participants belonging to these groups, resulting in low statistical power.

In two other studies, patients included in the group “non-Alzheimer dementia cases” were not further subcategorized. The information regarding the composition of this group was limited and therefore no specific conclusions about DLB cases can be made. Raszewski and colleagues concluded that HHcy is associated with an increased risk of non-Alzheimer’s dementia cases [80]. In addition, a significant relationship was found between Mini Mental State Examination (MMSE) scores and serum levels of Hcy only in the non-Alzheimer group [80]. The results of another prospective observational study indicated that there is a powerful connection between increased plasma Hcy levels and the risk of dementia of any type [25].

Lastly, compared with different dementia subtypes, DLB patients have an increased likelihood of experiencing affected nutritional parameters [81]. Thus, complications related to malnutrition may be prevented in the future by early interventions in all patients with dementia and especially in DLB patients. However, no difference was observed in terms of micronutrients including vitamin B12 and folate.

### 3.2. Tauopathies

Tauopathies are characterized by neuronal and glial inclusions which are formed by tau protein, a microtubule-binding protein [43]. Under normal circumstances, tau is present in the cytosol of neuronal and glial cells of the central nervous system and its main function is to stabilize the cell cytoskeleton by binding microtubules. In abnormal conditions of tauopathy, tau becomes hyperphosphorylated. Depending on which tau isoform is dominant in the cytoplasmic inclusions, tauopathies are classified as 3R, 4R or 3R:4R. CBD and PSP predominantly feature aggregates of the 4R-tau. By contrast, the 3R-tau form dominates in the aggregates of some other tau disorders, such as Pick disease. AD and frontotemporal lobar degeneration (FTLD) due to a microtubule-associated protein tau (MAPT) mutation feature both 3R and 4R tau inclusions. The shared tau protein pathology may explain why these diseases frequently overlap in terms of clinical symptoms and complex neuropathology.

Recent evidence supports the concept that elevated Hcy levels may be considered a metabolic risk factor for human tauopathies as Hcy appears to be implicated in the metabolism of the tau protein [23,82,83]. In particular, a study in a mouse model of tauopathy explored the effect of Hcy on tau aggregation and it has been showing that Hcy increased the levels of phosphorylated tau and enhanced its oligomerization and aggregation [82]. The same result of significant elevation of tau phosphorylation was observed in a subsequent study in a mouse model of tauopathy and HHcy [83]. In this study, synaptic pathology, astrocyte activation and worsening of behavioral deficits were observed as a result of elevated levels of brain Hcy [83]. In another mouse model of human tauopathy, decreased levels of cortical phosphorylated tau and alleviation of cognitive and motor deficits were observed following the dietary supplementation with L-methylfolate (the active folate form), choline and betaine [84]. Therapeutic administration of the above agents also led to a reduction in protein levels of Fyn, a tau tyrosine kinase that plays an important role in mediating tau-induced neurodegeneration, implicating that Hcy may modify tau-related proteins that further contribute to disease pathology [84]. Table 2 summarizes the results of the studies providing evidence of the imbalance of vitamins and Hcy in tauopathies, while Table S1 summarizes data regarding Hcy and vitamin dysregulation in neurodegenerative diseases other than PPS, in the included studies.

#### 3.2.1. Progressive Supranuclear Palsy

PSP, previously known as Steele–Richardson–Olszewski syndrome, is a rare parkinsonian syndrome characterized by oculomotor deficits, postural instability, akinesia and cognitive dysfunction. PSP is now recognized as encompassing several phenotypic syndromes. In particular, the Richardson syndrome represents the classical phenotype, while PSP with predominant parkinsonism, PSP with progressive gait freezing and PSP with predominant frontal presentation represent other common variants [86]. The clinical diagnostic criteria for PSP established by the Movement Disorder Society (MDS) are based on four functional domains with the aim of facilitating an early clinical diagnosis with increased specificity and sensitivity [87].

To the best of our knowledge, there are a few studies exploring the role of Hcy in PSP. Levin et al. included PD, PSP and Amyotrophic Lateral Sclerosis (ALS) patients in their study and revealed significantly increased concentrations of Hcy in these groups in comparison with the control subjects [85]. Moreover, apart from Hcy, MMA levels in the urine of PSP, PD and ALS patients were also increased to a significant extent and this observation implies that the whole metabolic pathway involving cysteine, MMA and Hcy is disturbed in the aforementioned neurodegenerative disorders. Whether Hcy is the cause, or a consequence of the neurodegeneration process remains to be investigated in further studies [85]. Another recent study that aimed to access the longitudinal change in metabolic status in patients with PSP showed that PSP patients experience a decrease in intake of total daily calories, proteins and vitamins (including folate) regardless of the presence of dysphagia [88]. In the study conducted by Chmiela et al., no statistically significant differences in Hcy levels have occurred between PD and PSP patients [59]. Moreover, in a cohort of patients with various neurodegenerative diseases, it was revealed that PD patients exhibited significantly decreased vitamin B12 levels measured within 3 years of neurological diagnosis compared with patients with PSP, FTD and DLB [15]. In contrast, vitamin B12 levels in PD patients were similar to those with either AD or MSA. Additionally, the rates of decline in vitamin B12 were greater in those with PD, AD and MCI, supporting the need for increased vigilance for vitamin B12 deficiency in these diseases [15]. Unfortunately, there was no comparison between vitamin B12 levels in these neurodegenerative conditions and healthy subjects.

#### 3.2.2. Corticobasal Degeneration

CBD is a heterogeneous disorder that encompasses different motor and cognitive phenotypes. This clinical heterogeneity has led to the use of the term “corticobasal syndrome” for cases with a clinical diagnosis, while the term “CBD” is reserved only for cases with neuropathologic confirmation. The most recent diagnostic criteria rely on expert consensus and identify different phenotypes associated with CBD pathology, with the aim to create two sets of consensus criteria for the disease: probable and possible CBD [89]. Core clinical characteristics for both categories include combinations of akinesia, rigidity, dystonia and myoclonus plus apraxia, cortical sensory loss and alien limb phenomena [89].

Apart from tau-related cytoskeletal pathology, TAR DNA-binding protein 43 (TDP-43) is also prominent in some CBD cases. In a study of 187 autopsy-confirmed CBD cases, TDP-43 pathology was found in almost 50 percent of the specimens, and those cases with a higher density of TDP-43 pathology clinically resembled a PSP phenotype [90]. It is important to report that hydroxocobalamin, a vitamin B12 analog, alleviated TDP-43 toxicity by reducing neuronal cell death induced by oxidative stress and mitochondrial dysfunction in mammalian neuronal cells and *Drosophila* models [91].

To our knowledge, there is a dearth of data investigating the association between the imbalance of vitamins, Hcy and CBD. In a study by Lovati et al. serum folate concentrations were obtained in patients with cortical and subcortical dementias [51]. Although mean folate levels did not differ significantly among patients with subcortical dementias (LBD, CBD, PSP, PD dementia) and controls, a tendency towards lower serum folate levels was observed in CBD and LBD patients in comparison to healthy subjects. Furthermore, CBD, FTD and AD groups exhibited the highest ratio of patients with serum folate levels below the reference range [51].

## 4. Conclusions and Future Directions

In this review, we aimed to sum up the available evidence concerning the involvement of Hcy and vitamins in the pathogenesis and development of PPS. These associations have been mainly studied in synucleinopathies and, to a lesser extent, in tauopathies. Hcy has been increasingly recognized as a potential culprit in neurodegeneration and many studies have shown that increased serum levels of Hcy can be found in syndromes with different disease pathologies. However, it must be noted that the levels of serum Hcy do not necessarily correlate with the brain levels of Hcy. As a result, direct assumptions should be made with caution. Conversely, some of the studies failed to show an imbalance of vitamins during the course of these disorders. The results of the available studies concerning the different levels of significance among the various syndromes are summarized in Table 3, while Table S2 summarizes the results of the available studies concerning the different levels of significance among Hcy, vitamins and neurodegenerative diseases other than PPS.

The inconsistency and variability of results across studies is not unexpected, given the different disease criteria being used, the lack of homogeneity in the collected samples and in the statistical methods applied and the relatively small sample sizes. Importantly, the sample sizes differ significantly between patients and healthy controls in some studies, making direct comparisons quite difficult to assess. It is obvious that more case–control studies for PSP and CBD and larger cohorts for MSA and DLB are needed in order to derive valuable data about Hcy elevation and vitamin insufficiency in PPS. It must be pointed out, that whether Hcy elevation in PPS is directly implicated in their pathophysiology or if it is due to a surrogate effect remains unclear. As we advance, it becomes more obvious that further prospective studies are essential to provide valuable insights regarding the association between Hcy levels and the duration of the disease for a causative relation to be established.

Acquiring data on the separate impact of elevated Hcy on different disease domains, e.g., cognitive function, parkinsonism and visual hallucinations would also be of value in terms of recognizing which domains are most affected by HHCy. In this regard, HHcy could predict more rapid cognitive dysfunction or rather motor progression among patients, thus modulating different phenotypes between PPS.

At present, the question of whether folate and vitamin B12 could be considered as symptomatic or even as disease-modifying treatments in PPS remains unanswered. With respect to future perspectives, interventional treatment studies using vitamins and assessing the effect of lowered Hcy on the clinical outcome of the patients are especially needed. In this way, the establishment of accurate guidelines and recommendations for vitamin B12 and folate supplementation in patients with PPS as well as the monitoring of these parameters would be more feasible to be achieved. In addition, it would be of interest to investigate the individualized effect of vitamin supplementation in different PPS, according to the distinct vitamin deficiency profile of each patient. Studies incorporating a broader range of responsible metabolic risk factors other than Hcy will further shed light on the unexplored underlying pathophysiologic mechanisms of these syndromes.

Regarding the hypothesis that a higher intake of folate or vitamin B12 could even prevent PD and related disorders, limited data exist. The results of a recent study provided moderate support for a possible protective effect of vitamin B12 on the development of PD. Yet, folate or vitamin B6 intake did not reduce the subsequent occurrence of PD [92]. Two more longitudinal studies failed to show any association between higher intake of folate and related B vitamins and the risk of PD, with a possible exception of vitamin B6 [93,94]. Further prospective studies in patients susceptible to suffering from PD and PPS will elucidate whether the evidence provided by in vitro and animal model studies [47,48] can be translated into clinically meaningful results.

Lastly, the multiple and complex effects of Hcy in degenerative disease processes suggest that vitamins implicated in its metabolism may be investigated in the future in the treatment approach of other degenerative disorders of the central nervous system.

## Figures and Tables

**Table 1:** Studies regarding Hcy and vitamin dysregulation in Parkinson plus syndromes associated with alpha-synuclein pathology.

First Author, Year, Country	Measurement Method	Diagnostic Criteria	Participants, Number	Sex (m/f)	Age (Mean ± SD)	Serum Homocysteine (Mean ± SD)	Serum Folate (Mean ± SD)	Serum Vitamin B12 (Mean ± SD)
Lovati, 2007 [51], Italy	Chemiluminescent immunoassay		HC, 76	18/58	67.6 ± 7.2	Not Measured	6.87 ± 3.50 μg/L	Not Measured
		The second International Workshop of the Consortium on DLB, 1999	LBD, 9	3/6	79.0 ± 4.0		4.26 ± 2.94 μg/L	
Zhang, 2015 [52], China	Not Mentioned	Consensus criteria for the clinical diagnosis of MSA (2008)	MSA, 161	82/79	57.99 ± 8.34	16.23 ± 8.09 μmol/L	6.46 ± 3.14 ng/mL	600.85 ± 515.69 pg/mL
			HC, 161	78/83	57.34 ± 10.37	14.04 ± 4.25 μmol/L	6.40 ± 3.28 ng/mL	547.1 ± 479.56 pg/mL
Chen, 2015 [53], China	Solid-phase competitive chemiluminescent enzyme immunoassay	Consensus criteria for the clinical diagnosis of MSA (1999)	MSA, 47	31/16	58.74 ± 10.18	13.28 ± 4.13 μmol/L	Not Measured	Not Measured
			HC, 50	27/23	55.64 ± 10.82	10.34 ± 3.07 μmol/L		
Zhou, 2016 [54], China	Not Mentioned	Consensus criteria for the clinical diagnosis of MSA (2008)	MSA, 55	37/18	59.33 ± 10.47	13.52 ± 4.56 μmol/L	Not Measured	Not Measured
			HC, 76	42/34	60.41 ± 11.50	10.28 ± 3.31 μmol/L		
Guo, 2017 [55], China	Routine laboratory tests	Consensus criteria for the clinical diagnosis of MSA (2008)	MSA, 53	30/23	59.7 ± 10.1	15.2 ± 5.95 μmol/L	Not Measured	Not Measured
			HC, 62	34/28	53.4 ± 9.08	10.4 ± 3.09 μmol/L		
Luthra, 2020 [15], USA	Chemiluminescent Assay	Not mentioned	DLB, 36	19/17	73 ± 8.6	Not Measured	Not Measured	518.7 ± 180.4 pg/mL
		Not mentioned	MSA, 9	6/3	65.1 ± 3.8			549.8 ± 145.6 pg/mL
Zhang, 2021 [56], China	Electrochemiluminescence immunoassays (ECLIA)	2017 DLB Diagnostic criteria	DLB, 132	64/68	72.7 ± 7.8	22.9 ± 16.3 μmol/L	8.0 ± 5.9 nmol/L	337.2 ± 210.4 pmol/L
			HC, 295	118/146	72.2 ± 6.1	13.0 ± 4.4 μmol/L	14.3 ± 8.0 nmol/L	457.5 ± 205.6 pmol/L
Hoffmann, 2021 [57], Germany	Electrochemiluminescence immunoassays (ECLIA)		HC, 54	13/41	72.00	16.45 * μmol/L	8.700 * ng/mL	415.0 * ng/L
		Not mentioned	LBD, 9	5/1	76.5	23.60 * μmol/L	5.950 * ng/mL	223.5 * ng/L
Song, 2022 [58], China	Enzymatic cycling assay		HC,62	34/28	61.40 ± 8.79	13.30 ± 6.29 μmol/L	10.99 ± 6.82 ng/mL	560.90 ± 3910.72 pg/mL
		2017 DLB Diagnostic criteria	LBD, 23	15/8	67.61 ± 7.74	16.60 ± 7.68 μmol/L	9.04 ± 5.23 ng/mL	342.00 ± 2640.94 pg/mL
Chmiela, 2022 [59], Poland	Not mentioned	Not mentioned	MSA, 14	8/6	65.2 ± 11.0	17.0 ± 8.9 μmol/L	Not Measured	Not Measured
Chen, 2023 [60], China	Electrochemistry method	Consensus criteria for the clinical diagnosis of MSA (2008)	MSA, 224	145/99	56.56 ± 7.55	Not measured	9.74 ± 5.71 μg/L	507.98 ± 339.80 ng/L
			HC, 244	145/99	56.06 ± 8.13		15.06 ± 5.25 μg/L	504.41 ± 217.30 ng/L

* Median values. DLB: Dementia with Lewy Bodies, LBD: Lewy Body Dementia, HC: Healthy Controls, MSA: Multiple System Atrophy.

**Table 2:** Studies regarding Hcy and vitamin dysregulation in Parkinson plus syndromes associated with tau pathology.

First Author, Year, Country	Measurement Method	Diagnostic Criteria	Participants, Number	Sex (m/f)	Age (Mean ± SD)	Serum Homocysteine (Mean ± SD)	Serum Folate (Mean ± SD)	Serum Vitamin B12 (Mean ± SD)
Lovati, 2007 [51], Italy	Chemiluminescent immunoassay		HC, 76	18/58	67.6 ± 7.2	Not Measured	6.87 ± 3.50 μg/L	Not Measured
		Accuracy of the clinical diagnosis of corticobasal degeneration, 1997	CBD, 5	3/2	69.6 ± 9.6		4.30 ± 2.80 μg/L	
		Clinical research criteria for the diagnosis of PSP, 1996	PSP, 6	3/3	71.8 ± 4.8		5.74 ± 4.05 μg/L	
Levin, 2010 [85], Germany	Automated ligand- binding assays	Clinical and PET diagnostics	PSP, 22	12/10	66.95 ± 5.71	15.75 ± 5.75 μmol/L	Not Mentioned	Not Mentioned
			HC, 30	13/12	63.62 ± 11.32	11.19 ± 1.21 μmol/L		
Luthra, 2020 [15], USA	Chemiluminescent Assay	Not mentioned	PSP, 20	11/9	69 ± 6.1	Not Measured	Not Measured	539.1 ± 178.1 pg/mL
Chmiela, 2022 [59], Poland	Not mentioned	Not mentioned	PSP, 33	15/18	70.1 ± 7.1	13.8 ± 3.4 μmol/L	Not Measured	Not Measured

CBD: Corticobasal Degeneration, HC: Healthy Controls, PSP: Progressive Supranuclear Palsy.

**Table 3:** Summarized results of the available studies concerning the different levels of significance among the various Parkinson plus syndromes and Hcy and vitamins.

First Author, Year, Country	All Participants	PPS Participants	Level of Significance Hcy between Disease and HC	Level of Significance Folic between Disease and HC	Level of Significance B12 between Disease and HC	Level of Significance Hcy between Diseases	Level of Significance Folic between Diseases	Level of Significance B12 between Diseases
Lovati, 2007 [51], Italy	HC, AD, FTD, LBD, CBD, PSP, PD-d	LBD	Not Measured	Not Significant	Not Measured	Not Measured	Cortical (AD, FTD) vs. subcortical (LBD, CBD, PSP, PD-d) dementias: *p* < 0.01	Not Measured
		CBD		Not Significant				
		PSP		Not Significant				
Levin, 2010 [85], Germany	PD, PSP, ALS, HC	PSP	*p* < 0.001	Not Significant	Not Significant	Levodopa- treated PD patients vs. patient groups with other neurodegenerative diseases (PSP, ALS): Not Significant	Not Reported	Not Reported
Zhang, 2015 [52], China	MSA, HC	MSA	*p* < 0.05	Not Significant	Not Significant	—	—	—
Chen, 2015 [53], China	MSA, PD, HC	MSA	*p* = 0.005	Not Measured	Not Measured	MSA vs. PD: *p* = 0.897	Not Measured	Not Measured
Zhou, 2016 [54], China	MSA, HC	MSA	*p* < 0.001	Not Measured	Not Measured	-	Not Measured	Not Measured
Guo, 2017 [55], China	MSA, PD, HC	MSA	*p* = 0.005	Not Measured	Not Measured	MSA vs. PD: *p* = 0.865	Not Measured	Not Measured
Luthra, 2020 [15], USA	AD, DLB, FTD, MCI, MSA, PD, PSP	DLB	Not Measured	Not Measured	Not Measured	Not Measured	Not Measured	PD vs. either AD or MSA: Not Significant All disease groups (AD, DLB, FTD, MCI, MSA, PD, PSP) *p* = 0.015
		MSA						
		PSP						
Zhang, 2021 [56], China	DLB, AD, HC	DLB	*p* < 0.01	*p* < 0.01	*p* < 0.01	DLB vs. AD: *p* < 0.05	DLB vs. AD: Not Significant	DLB vs. AD: *p* < 0.05
Hoffmann, 2021 [57], Germany	FTD, LBD, AD, VaD, HC	LBD	*p* = 0.1390	*p* = 0.0773	*p* = 0.0207	All disease groups (FTD, LBD, AD, VaD): Not Significant	All disease groups (FTD, LBD, AD, VaD): Not Significant	All disease groups (FTD, LBD, AD, VaD): Not Significant
Song, 2022 [58], China	HC, MCI, AD, VaD, FTD, LBD	LBD	*p* < 0.01	Not Significant	*p* < 0.01	All disease groups (MCI, AD, VaD, FTD, LBD): *p* < 0.001	All disease groups (MCI, AD, VaD, FTD, LBD): *p* = 0.330	All disease groups (MCI, AD, VaD, FTD, LBD): *p* < 0.001
Chmiela, 2022 [59], Poland	PD, MSA, PSP	MSA	Not Measured	Not Measured	Not Measured	All disease groups (PD, MSA, PSP) *p* = 0.402	Not Measured	Not Measured
		PSP						
Chen, 2023 [60], China	MSA, PD, HC	MSA	Not Measured	<0.001	0.083	Not Measured	MSA vs. PD 0.502	MSA vs. PD 0.181

AD: Alzheimer’s Disease, ALS: Amyotrophic Lateral Sclerosis, CBD: Corticobasal Degeneration, DLB: Dementia with Lewy Bodies, FTD: Frontotemporal Dementia, HC: Healthy Controls, MCI: Mild Cognitive Impairment, MSA: Multiple System Atrophy, PD: Parkinson’s Disease, PD-d: Parkinson Disease Dementia, PSP: Progressive Supranuclear Palsy, VaD: Vascular Dementia.

## Supplementary Materials

The following supporting information can be downloaded at https://www.mdpi.com/article/10.3390/biom14101213/s1. Table S1: Data regarding Hcy and vitamin dysregulation in neurodegenerative diseases other than PPS in the included studies; Table S2: Summarized results of the available studies concerning the different levels of significance among Hcy, vitamins and neurodegenerative diseases other than PPS.
