# Supplementary material for: The Imbalance of Homocysteine, Vitamin B12 and Folic Acid in Parkinson Plus Syndromes: A Review beyond Parkinson Disease
Source: Biomolecules. 2024 Sep 26;14(10):1213. doi: 10.3390/biom14101213 (PMC11506381; doi:10.3390/biom14101213)
Supplement: Supplementary file 1 [file biomolecules-14-01213-s001.zip › biomolecules-3104114-supplementary.pdf]

biomolecules

MDPI

Table S1. . Data regarding Hcy and vitamins dysregulation in neurodegenerative diseases other than PPS, in the included studies.

First Author, Year, Country	Measurement Method	Diagnostic criteria	Participants, Number	Sex (m/f)	Age (Mean±SD)	Serum Homocysteine (Mean±SD)	Serum Folate (Mean±SD)	Serum Vitamin B12 (Mean±SD)
Lovati, 2007 [51], Italy	Chemiluminescent immunoassay		HC, 76	18/58	67.6±7.2	Not Measured	6.87±3.50 µg/L	Not Measured
		NINCDS-ADRDA criteria	AD, 108	27/81	76.6±7.5		3.62±2.35 µg/L	
		Consensus clinical diagnostic criteria, 1998	FTD, 28	13/15	71.5±7.2		3.52±1.71 µg/L	
		Parkinson disease neuropathology: later-developing dementia and loss of the levodopa response, 2002	PD-Dementia, 37	17/20	68.4±11.2		5.73±2.74 µg/L	
Levin, 2010 [85], Germany	Automated ligand-binding assays	Clinical & electrophysiological criteria	ALS, 27	19/8	66.04±7.45	13.94±3.47 µmol/l	Not Mentioned	Not Mentioned
		UK Brain Bank criteria	PD, 41	17/14	66.29±7.69	15.05± 4.63 µmol/l		
			HC, 30	13/12	63.62±11.32	11.19± 1.21 µmol/l		
Chen, 2015 [53], China	Solid-phase competitive chemiluminescent enzyme immunoassay	United Kingdom PD Society Brain Bank (UK-PDSBB) criteria 1992	PD, 60	34/26	63.10±10.62	12.89 ± 5.70 umol/l	Not Measured	Not Measured
			HC, 50	27/23	55.64±10.82	10.34±3.07 umol/l		

Guo, 2017 [55], China	Routine laboratory tests	United Kingdom PD Society Brain Bank (UK-PDSBB) criteria 1992	PD, 65	38/27	61.5±7.97	14.3±5.76 μmol/L	Not Measured	Not Measured
			HC, 62	34/28	53.4±9.08	10.4±3.09 μmol/L		
Luthra, 2020 [15], USA	Chemiluminescent Assay	Not mentioned	AD, 204	86/118	73.8±9.7	Not Measured	Not Measured	465.1±165.5 pg/ml
		Not mentioned	FTD, 24	13/11	67.7± 8.5			542.9±159.3 pg/ml
		Not mentioned	MCI, 290	140/150	69.8±9.9			499.4±180.7 pg/ml
		Not mentioned	PD, 130	79/51	68.4±9.7			454.7±168.4 pg/ml
Zhang, 2021 [56], China	Electrochemiluminescence immunoassays (ECLIA)	NINCDS-ADRDA criteria	AD, 264	145/150	71.9±8.6	18.4±11.6 μmol/l	8.4±9.1 nmol/l	397.0±213.9 pmol/l
			HC, 295	118/146	72.2±6.1	13.0±4.4 μmol/l	14.3±8.0 nmol/l	457.5±205.6 pmol/l
Hoffmann, 2021 [57], Germany	Electrochemiluminescence immunoassays (ECLIA)		HC, 54	13/41	72.00	16.45* μmol/L	8.700* ng/mL	415.0* ng/L
		Not mentioned	FTD, 9	6/3	71.56	15.35* μmol/L	6.700* ng/mL	262.5* ng/L
		Not mentioned	AD, 42	15/27	78.83	32.00* μmol/L	6.550* ng/mL	248.0* ng/L
		Not mentioned	VaD, 26	12/14	78.19	21.20* μmol/L	7.900* ng/mL	325.0* ng/L
Song, 2022 [58], China	Enzymatic cycling assay		HC, 62	34/28	61.40±8.79	13.30±6.29 μmol/L	10.99±6.82 ng/mL	560.90±3910.72 pg/ml
		NIA-AA criteria	AD, 64	30/34	65.08±6.38	16.50±5.83 μmol/L	8.26±4.74 ng/mL	314.28±2550.67 pg/ml
		International behavioral variant FTDC criteria	FTD, 48	20/28	62.10±6.12	15.80±6.07 μmol/L	8.28±5.26 ng/mL	409.38±3220.04 pg/ml
		Modified Petersen's criteria	MCI, 36	16/20	63.22±7.27	13.86±5.81 μmol/L	9.25±4.58 ng/mL	464.94±3650.15 pg/ml
		NINDS-AIREN	VaD, 24	18/6	65.96±7.22	17.85±7.73 μmol/L	9.44±6.93 ng/mL	333.75±2740.74 pg/ml

		Work Group Criteria						
Chmiela, 2022 [59], Poland	Not mentioned	Not mentioned	PD, 304	180/124	65.3±9.8	16.3±7.9 umol/l	Not Measured	Not Measured
Chen, 2023 [60], China	Electrochemistry method	MDS Clinical Diagnostic Criteria for Parkinson's Disease 2015	PD, 220	113/87	57.26±7.82	Not Measured	10.17±6.09 μg/L	488.00±388.81 ng/L
			HC, 244	145/99	56.06±8.13		15.06±5.25 μg/L	504.41±217.30 ng/L

*Median values

AD: Alzheimer's Disease, ALS: Amyotrophic Lateral Sclerosis, FTD: Frontotemporal Dementia, HC: Healthy Controls, MCI: Mild Cognitive Impairment, PD-d: Parkinson Disease-Dementia, VaD: Vascular Dementia

Table S2. Summarized results of the available studies concerning the different levels of significance among Hcy, vitamins and neurodegenerative diseases other than PPS.

First Author, Year, Country	Participants with other neurodegenerative diseases	Level of significance Hcy between disease and HC	Level of significance folic between disease and HC	Level of significance B12 between disease and HC
Lovati, 2007 [51], Italy	AD	Not Measured	p<0.001	Not Measured
	FTD		p<0.001	
	PD-d		Not Significant	
Levin, 2010 [85], Germany	PD	p<0.001	Not Significant	Not Significant
	ALS	p < 0.001	Not Significant	Not Significant
Zhang, 2021 [56], China	AD	p<0.01	p<0.01	p<0.01
Hoffmann, 2021 [57], Germany	VaD	p=0.2613	p=0.4896	p=0.0535
	FTD	p=0.7033	p=0.2796	p=0.0549
	AD	p=0.0399	p=0.0281	p=0.0006
Song, 2022 [58], China	AD	p<0.001	Not Significant	p<0.001
	FTD	p<0.001	Not Significant	p<0.01
	MCI	Not significant	Not Significant	Not Significant

	VaD	p<0.001	Not Significant	p<0.01
Chen, 2023 [60], China	PD	Not Measured	<0.001	<0.001

AD: Alzheimer's Disease, ALS: Amyotrophic Lateral Sclerosis, FTD: Frontotemporal Dementia, HC: Healthy Controls, MCI: Mild Cognitive Impairment, PD: Parkinson's Disease, PD-d: Parkinson Disease-Dementia, VaD: Vascular Dementia
